# Association of matrix metalloproteinase family gene polymorphisms with lung cancer risk: logistic regression and generalized odds of published data

**DOI:** 10.1038/srep10056

**Published:** 2015-07-22

**Authors:** Hongxia Li, Xiaoyan Liang, Xuebing Qin, Shaohua Cai, Senyang Yu

**Affiliations:** 1 Department of Respiratory Medicine, South Building, Chinese PLA General Hospital, Beijing 100853; 2 Department of Respiratory Medicine, Special Inpatient Unit, Chinese PLA General Hospital, Beijing 100853

## Abstract

Many studies have reported the association between the matrix metalloproteinase (MMP) polymorphisms and lung cancer susceptibility, but the results were inconclusive. We conducted a meta-analysis, using a comprehensive strategy based on the logistic regression and a model-free approach, to derive a more precise estimation of the relationship between MMP1, MMP2, MMP9 and MMP13 polymorphisms with lung cancer risk. A total of 22 case-control studies including 8202 cases and 7578 controls were included in this meta-analysis. For MMP1-1607 1G/2G, increased lung cancer risk was found among Asians in additive model(OR = 1.34, 95%CI:1.18-1.53) and with model-free approach(OR_G_ = 1.41, 95%CI:1.21-1.65). For MMP2-1306 C/T and -735 C/T, based on the model-free approach, a significantly reduced risk was found in Asians(MMP2-1306 C/T:OR_G_ = 0.49,95%CI:0.42-0.57; MMP2-735 C/T: OR_G_ = 0.71, 95%CI:0.61-0.84). For MMP9-1562 C/T, a significantly increased risk was found among Asians(OR = 2.73, 95%CI:1.74-4.27) with model-free approach. For MMP13-77A/G, there was no association between this polymorphism and lung cancer risk in the recessive model(OR = 1.02, 95%CI:0.83-1.26) and with the model-free approach(OR_G_ = 0.95, 95%CI:0.76-1.17). Therefore, this meta-analysis suggests that the MMP1-1607 1G/2G, MMP2-1306 C/T, MMP2-735 C/T, MMP9 -1562 C/T polymorphisms were risk factors for lung cancer among Asians, while MMP13 -77A/G polymorphism was not associated with lung cancer risk.

Lung cancer is the leading cause of cancer-related death worldwide and responsible for approximately 1.3 million deaths each year^1^. Despite the great progress made in several areas of oncology, the prognosis of lung cancer remains dismal^2^. The exact cause and mechanism of lung cancer are still under investigation. Epidemiological studies have demonstrated tobacco smoking as well as exposure to environmental tobacco smoke in healthy non-tobacco users as the major risk factor for lung cancer^3^. However, not all smokers develop lung cancer and a fraction of life long non-smokers will die from lung cancer indicating that genetic factors may play a significant role in determining the susceptibility to lung cancer^3^,^4^.

The matrix metalloproteases (MMPs) are zinc-dependent endopeptidases that degrade the extracellular matrix collagens and belong to a larger family of proteases known as the metzincin superfamily. 5,6 Matrix metalloproteinase-1 (MMP-1) may degrade a broad range of substrates including the interstitial types I, II, III collagens as well as casein and contribute to tumor growth and spread by altering the cellular microenvironment to favor tumor formation. 5,6,7,8 Among secreted MMPs, MMP-2 and MMP-9 are known to play a major role in cancer invasion and metastasis development by their ability to degrade type IV collagen^9^. Furthermore, overexpression of MMP13 has been related to more aggressive tumors and poor prognosis in lung cancer^10^,^11^.

Polymorphisms in the regulatory regions of MMPs have been associated with changes in the expression level of these genes in different human cancer^12^,^13^,^14^. Up to now, many molecular epidemiological studies have investigated the association between the MMPs polymorphism and lung cancer risk^15^,^16^,^17^,^18^,^19^,^20^,^21^,^22^,^23^,^24^,^25^,^26^,^27^,^28^,^29^,^30^,^31^. However, the results remain controversial and ambiguous. Several meta-analysis have been performed to assess MMPs polymorphism in lung cancer, but these analyses are mainly based on traditional approaches, which would lead to multiple comparisons or erroneous mode specification without prior biological evidence. Therefore, we conducted this meta-analysis based 22 case-control studies by a comprehensive statistical strategy of a logistic regression and a model-free approach^32^,^33^.

## Materials and Methods

### Search Strategy

We searched for relevant studies up to May 2014 through the PubMed, Embase, Wanfang (http://www.wanfangdata.com.cn), China National Knowledge Infrastructure Platform (CNKI; http://www.cnki.net) database with the following terms and their combinations: “lung cancer/carcinoma”, “polymorphism/variant”, and “metalloproteinase/MMP”. We tried to identify potentially relevant studies from the whole reference lists by orderly reviewing title, abstract and full text.

### Selection criteria

The inclusion criteria were as follows: a) case-control studies focused on the association of MMP1, MMP2, MMP9 or MMP13 polymorphism and lung cancer; b) genotype and allele data available. Studies were excluded for following reasons: a) unpublished papers, reviews and duplication of publications; b) data unavailable for calculating genotype or allele frequencies; c) no control population. Additionally, investigations of departure from Hardy-Weinberg equilibrium (HWE) was excluded from the final analysis. If more than one article was published using the same case series, we selected the study with the largest sample size.

### Data extraction

All the available data were extracted from each study by two investigators (H X L and X Y L) independently according to the inclusion criteria listed above. For each study, we recorded the first author, year of publication, country of origin, ethnicity, the method of genotyping, the number of cases and controls and genotype distributions in cases and controls.

### Statistical analysis

Hardy-Weinberg equilibrium was examined by chi-square goodness-of-fit test (*P* > 0.05) using gene frequencies of the healthy individuals. Metagen (http://bioinformatics.biol.uoa.gr/~pbagos/metagen/) was used by selecting the genetic model. Two parameters, θ2 and θ3, were calculated using the formula: log it (πij) = α_i_ + θ_2_z_i2_ + θ_3_z_i3_ and OR _AB/AA_ = exp (θ_2_), OR _BB/AA_ = exp (θ_3_); where α_i_ is the indicator of study-specific fixed-effect; θ_2_ and θ_3_ are dummy variables of genotypes AB and BB. The appropriate genetic model was identified using the following criteria:(i) No association: θ_2_ = θ_3_; (ii) Dominant model: θ_2_ = θ_3_ > 0; (iii) Recessive model: θ_2_ = 0 and θ_3_ > 0; (iv) Additive model: 2θ_2_ = θ_3_; (v) Co-dominant model: θ_3_ > θ_2_ > 0; (vi) Complete overdominant model: θ_2_ > 0 and θ_3_ = 0. Finally, once the most appropriate genetic model was identified, the pooled OR with corresponding 95% confidence interval (95% CI) was estimated in logistic regression model. Additionally, Zintzaras reported a novel method to calculate the generalized odds ratio (OR_G_) based on a genetic model-free approach, which may overcome the short-comings of multiple model testing or erroneous model specification^33^. Thus, the OR_G_ calculations were also performed.

The heterogeneity of the studies was assessed using the Cochran’s Q test (considered significant for P < 0.10) and was quantified by the *I*^*2*^ statistic. Both fixed effects (Mantel-Haenszel) and random effects (Der Simonian and Laird) models were used to combine the data. Relative influence of each study on the pooled estimate was assessed by omitting one study at a time for sensitivity analysis. Publication bias was evaluated by visual inspection of symmetry of Begg’s funnel plot and assessment of Egger’s test (P < 0.05 was regarded as representative of statistical significance). Statistical analyses were done in ORGGASMA, metan and metagen in STATA software, version 11.0 (STATA Corp., College Station, TX, USA), and all tests were two-sided.

## Results

### Characteristics of the studies

**Figure 1:**
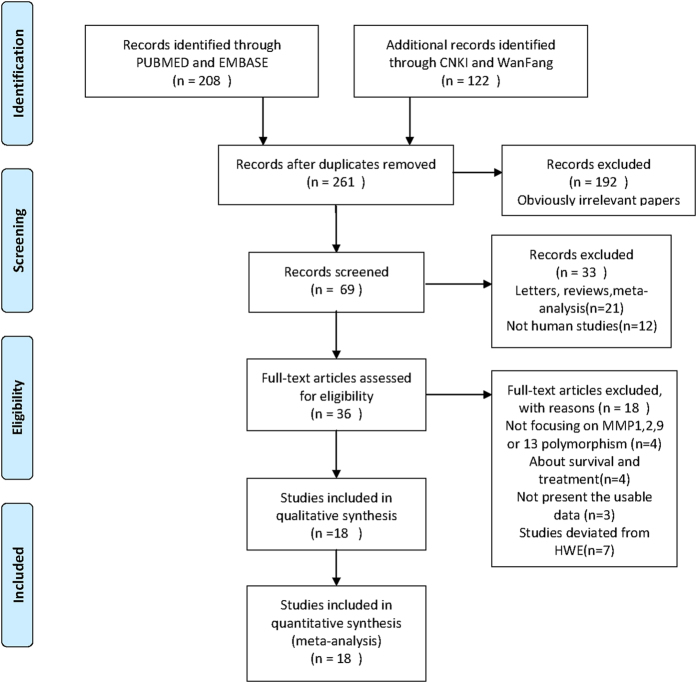
Flow diagram of studies identification.

There were 330 papers relevant to the search words. The flow chart of selection of studies and reasons for exclusion is presented in Fig. 1. Overall, 18 publications with 22 case-control studies including 8202 cases and 7578 controls were available for this analysis. Seven studies with 3996 cases and 3507 controls for MMP1-1607 1G/2G polymorphism, five studies with 2004 cases and 1967 controls for MMP2-1306 C/T polymorphism, three studies with 1229 cases and 1303 controls for MMP2-735 C/T polymorphism, four studies with 1202 cases and 1039 controls for MMP9-1562 C/T polymorphism, and three studies with 1221 cases and 1225 controls for MMP13-77A/G polymorphism. Study characteristics are summarized in Table 1. The genotype distributions in the controls of all studies were consistent with HWE.

### Quantitative synthesis

**Figure 2:**
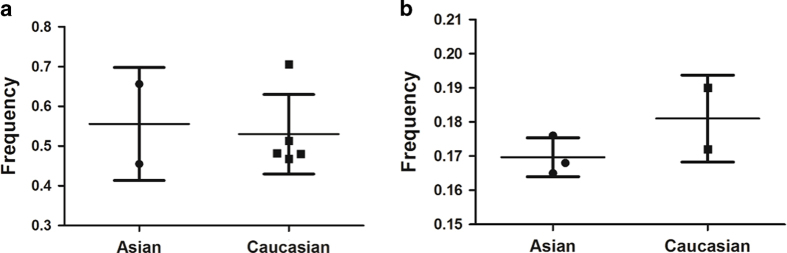
Frequencies of the variant alleles among control subjects stratified by ethnicity. (**a**) MMP1 -1607 2G allele; (**b**) MMP2 -1306 T allele.

There was a variation in the 2G allele frequency of the MMP1-1607 1G/2G polymorphism among the controls across different ethnicities, ranging from 0.46 to 0.71. For Asian controls, the 2G allele frequency was 0.56, which was slightly higher than that in Caucasian controls (0.53, P = 0.791; Fig. 2A). Another variation was in the T allele frequency of the MMP2-1306 C/T polymorphism among the controls across different ethnicities, ranging from 0.17 to 0.19. For Asian controls, the T allele frequency was 0.17, which was slightly lower than that in Caucasian controls (0.18, P = 0.249; Fig. 2B).

**Figure 3:**
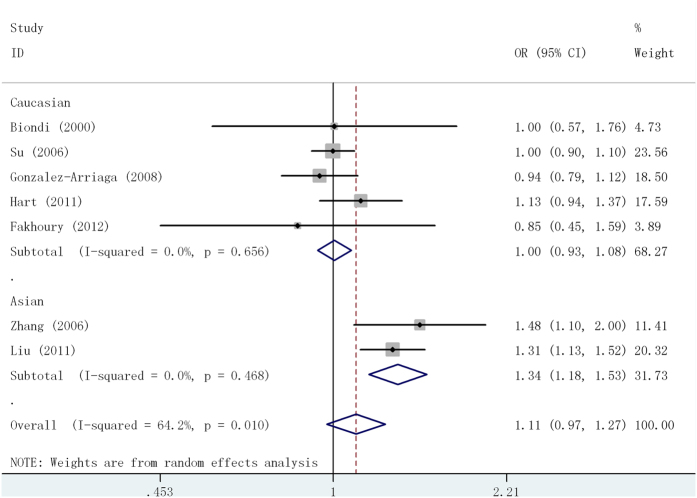
Odds ratios (OR) and 95% confidence interval (CI) of individual studies and pooled data for the association of MMP1-1607 1G/2G polymorphism and lung cancer risk in additive model.

Five common SNPs occurred in MMP1, MMP2, MMP9 and MMP13 sequences were included in the quantitative synthesis, and detail results were shown in Table 2. For the MMP1 -1607 1G/2G polymorphism, the pooled OR_1G2G/1G1G_ and OR_2G2G/1G1G_ were 1.08(95%CI = 0.96-1.21) and 1.16(95%CI = 1.02-1.33), respectively, suggesting an additive model was assessed using traditional method. Overall, no significant association with lung cancer risk was detected for MMP1 -1607 1G/2G polymorphism in additive model and heterogeneity between studies was observed in the overall comparison. In subgroup analysis based on ethnicity, however, the heterogeneity disappeared and a significantly increased risk was found in Asians(OR = 1.34, 95%CI:1.18-1.53) (Fig. 3). Based on the model-free approach, significant result was also produced for MMP1 -1607 1G/2G polymorphism and lung cancer risk among Asians(OR_G_ = 1.41, 95%CI:1.21-1.65). No significant association was found in subgroup analyses based on the source of control and sample size (Table 2).

**Figure 4:**
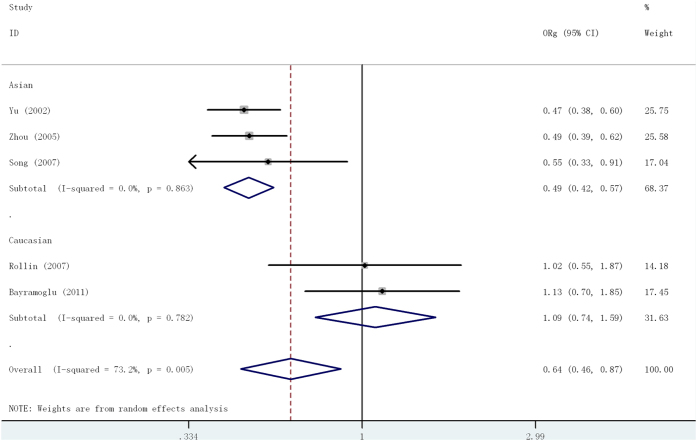
Forest plot of the generalized odds ratio (OR_G_) and 95% confidence intervals (CIs) of studies on the association between lung cancer and the MMP2-1306 C/T polymorphism based on model-free approach.

For the MMP2 -1306 C/T polymorphism, the pooled OR_CT/CC_ and OR_TT/CC_ were 0.54(95%CI = 0.47-0.63) and 0.53(95%CI = 0.33-0.85), respectively, suggesting no appropriate genetic model was assessed using traditional method. Based on the model-free approach, significant result was found in the overall comparison (OR_G_ = 0.64, 95%CI:0.46-0.87) and among Asians (OR_G_ = 0.49, 95%CI:0.42-0.57), but not among Caucasians(OR_G_ = 1.09, 95%CI:0.74-1.59) (Fig. 4). Stratified by source of control, a significantly risk was found in the population-based studies, however, no significant association was observed in the hospital-based studies (Table 2). When stratifying by sample size, a significant association was found in sample size ≥ 500 studies (Table 2). No significant heterogeneity between studies was observed in subgroup analyses.

For the MMP2 -735 C/T polymorphism, the pooled OR_CT/CC_ and OR_TT/CC_ were 0.70(95%CI = 0.59-0.83) and 0.75(95%CI = 0.51-1.10), respectively, suggesting no appropriate genetic model was assessed using traditional method. Based on the model-free approach, significant result was found in the overall comparison (OR_G_ = 0.72, 95%CI:0.62-0.84) and among Asians(OR_G_ = 0.71, 95%CI:0.61-0.84), but not among Caucasians(OR_G_ = 0.85, 95%CI:0.44-1.67). Stratified by source of control, a significantly risk was found in the population-based studies, however, no significant association was observed in the hospital-based studies (Table 2). When stratifying by sample size, a significant association was found in sample size ≥ 500 studies (Table 2). No significant heterogeneity between studies was observed in the overall comparisons as well as in subgroup analyses.

For the MMP9 -1562 C/T polymorphism, the pooled OR_CT/CC_ and OR_TT/CC_ were 1.14(95%CI = 0.70-1.87) and 0.46(95%CI = 0.11-2.00), respectively, suggesting a complete overdominant model was assessed using traditional method. Overall, no significant association with lung cancer risk was detected for MMP9 -1562 C/T polymorphism in complete overdominant model and heterogeneity between studies was observed in the overall comparison. In subgroup analysis based on ethnicity, however, a significantly decreased risk was found in Asians(OR = 0.40, 95%CI:0.25-0.64), suggesting homozygotes were at a lesser risk of lung cancer than heterozygotes. Based on the model-free approach, significant result was also found in Asians(OR_G_ = 2.73, 95%CI:1.74-4.27), suggesting lung cancer cases with higher mutational load than healthy individuals have higher risk for lung cancer susceptibility. Stratified by source of control, a significantly risk was found in the population-based studies, however, no significant association was observed in the hospital-based studies (Table 2). When stratifying by sample size, no significant association was found (Table 2). Heterogeneity between studies was observed in the overall comparisons and subgroup analysis based on sample size, but not in subgroup analysis based on ethnicity.

**Figure 5:**
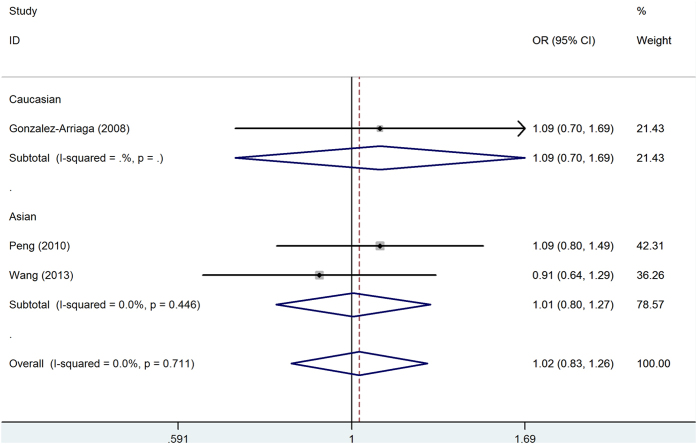
Forest plot of the generalized odds ratio (OR_G_) and 95% confidence intervals (CIs) of studies on the association between lung cancer and the MMP13-77 A/G polymorphism in recessive model.

For the MMP13 -77A/G polymorphism, the pooled OR_AG/AA_ and OR_GG/AA_ were 0.99(95%CI = 0.83-1.18) and 1.32(95%CI = 1.03-1.67), respectively, suggesting a recessive model was assessed using traditional method. In recessive model, no significant association with lung cancer risk was detected for MMP13 -77A/G polymorphism in overall comparison and subgroup analysis (Fig. 5). Based on the model-free approach, no significant result was also found in overall comparison and subgroup analysis (Table 2).

### Sensitive analysis

**Figure 6:**
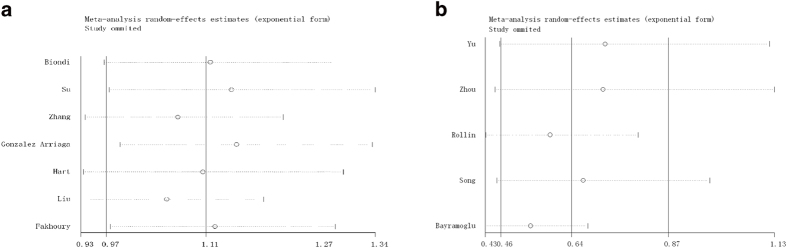
Sensitivity analysis: examining the influence of individual studies to pooled odds ratios (OR). (**a**) MMP1-1607 1G/2G polymorphism in additive model; (**b**) MMP2-1306 C/T polymorphism for generalized odds ratio (OR_G_).

Sensitivity analyses were performed to assess the influence of individual dataset on the pooled ORs by sequential removing each eligible study. As seen in Fig. 6, any single study was omitted, while the overall statistical significance does not change, indicating that our results are statistically robust.

### Publication bias

**Figure 7:**
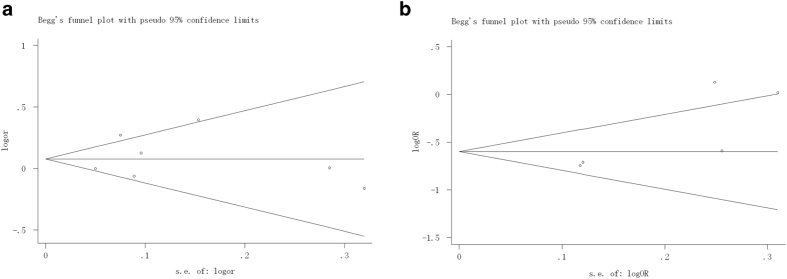
Begg’s funnel plot for publication bias test. Each point represents a separate study for the indicated association. (**a**) Funnel plot for additive model of MMP1-1607 1G/2G polymorphism; (**b**) Funnel plot for generalized odds ratio (OR_G_) of MMP2-1306 C/T polymorphism.

Begg’s funnel plot and Egger’s test were performed to assess publication bias among the literatures. As shown in Fig. 7, there was no evidence of publication bias for MMP1 -1607 1G/2G in additive model (Begg’s test *P* = 1.000; Egger’s test *P* = 0.703) and MMP2 -1306 C/T in generalized odds ratio (Begg’s test *P* = 0.221; Egger’s test *P* = 0.076).

## Discussion

Meta-analysis is a powerful statistical tool to resolve the discrepancies across individual studies by integrating existing published data. At present, the majority of meta-analyses of genetic association studies are usually conducted by comparing genotype frequencies between cases and controls under various genetic models. However, these genetic models are not independent, and a priori knowledge or biological justification for model selection is seldom available^34^,^35^. Therefore, we performed this meta-analysis about MMP1, 2, 9 and 13 polymorphisms and lung cancer risk by a comprehensive strategy, including logistic regression and model-free approach^32^,^33^, to avoid erroneous model specification and multiple model tests with the risk of an inflated Type I error rate.

In the current study, a total of 22 case-control studies with 8202 cases and 7578 controls were included in the meta-analysis^12^,^15^,^16^,^17^,^18^,^19^,^20^,^21^,^22^,^23^,^24^,^25^,^26^,^27^,^28^,^29^,^30^,^31^, and the association between MMP1-1607 1G/2G, MMP2-1306 C/T, MMP2-735 C/T, MMP9 -1562 C/T and MMP13 -77A/G polymorphisms and lung cancer risk was explored. Our results suggest that MMP1-1607 1G/2G, MMP2-1306 C/T, MMP2-735 C/T, MMP9 -1562 C/T polymorphisms were significantly associated with lung cancer risk among Asian population, but there is no association found between MMP13 -77A/G polymorphism and susceptibility to lung cancer.

This finding may be biologically plausible. MMPs play roles in many important physiological and pathological processes including cancer and lung inflammation through degradation of basal membranes and extracellular matrix^24^,^25^,^36^. Expression of MMPs has been linked to a wide range of cancer types including lung cancer and has been reported to be correlated with tumor invasion and poor prognosis^24^,^25^,^26^,^27^. In recent years, several SNPs (MMP1-1607 1G/2G, MMP2-1306 C/T, MMP2-735 C/T, MMP9 -1562 C/T and MMP13 -77A/G) in the promoter region of the MMP genes have been reported^26^,^27^,^28^,^31^. Functional analyses of these polymorphisms in MMP genes have found their modulatory effect on transcriptional activity, leading to alterations in the gene expression^13^,^14^,^36^,^37^. For MMP1-1607 1G/2G polymorphism, the promoter with the 2G allele has significantly stronger transcriptional activity compared with the 1G promoter, because the 2G allele creates a transcription factor binding site and increases transcription capacity^38^. It has been demonstrated that individuals with CC genotype of both MMP2 -735 C/T and -1306 C/T polymorphisms have higher promoter activity and higher MMP-2 enzyme activity compared with those with the TT genotype, and thus may have potentially higher risk of lung cancer^39^,^40^. The MMP9 -1562 C to T substitution has been shown to up-regulate the promoter activity and the presence of the -1562T allele has also been found to associated with the decease of the capacity of a putative transcription repressor protein with a subsequent increase in gene expression^41^. Results from six independent transfection experiments *in vitro* with MMP13 -77A/G constructs indicated that the constructs with A had about twice as much transcriptional activity as the constructs with G in the same location^37^. It has been suggested that these SNPs are associated with the development of different human cancer^13^,^14^,^15^,^16^,^42^,^43^.

MMP polymorphisms and lung cancer risk have been investigated by several meta-analyses^44^,^45^,^46^,^47^. Recently, Hu *et al* conducted a comprehensive meta-analysis about five MMP polymorphisms and lung cancer susceptibility, and found that the MMP1-1607 1G/2G and MMP2-1306 C/T confer significantly susceptibility to lung cancer, and MMP1-1607’s effect was dependent on ethnicity, consistent with the results of this meta-analysis^44^. Compared with Hu’s work, we excluded three studies deviating from Hardy-Weinberg equilibrium(HWE)^7^,^48^,^49^, identified more eligible studies^21^,^28^,^29^,^30^ and performed a detailed analysis by logistic regression and model-free approach. We also found some significant associations that were not observed in Hu’s study, one example of which was that we found the MMP2 -735 C/T decreased lung cancer risk for Asians, whereas no significant result was found for Caucasians. On the other hand, we also analyzed the MMP13 -77A/G polymorphism. Compared with several other meta-analysis about MMP polymorphisms and lung cancer risk reported by Guo XT *et al*^45^, Hu J *et al *46 and Wang J *et al *47, we identified more eligible studies, evaluated more SNPs(MMP1-1607 1G/2G, MMP2-1306 C/T, MMP2-735 C/T, MMP9 -1562 C/T and MMP13 -77A/G) and performed analysis by a comprehensive strategy, while they only analyzed single polymorphism and lung cancer risk.

Some heterogeneity factors between studies that could limit the strengths of the meta-analysis should be addressed. First, ethnicity was one of the most important factors that could lead to heterogeneity because of the diverse genetic backgrounds and environmental factors in different ethnicities. Second, the source of the controls was another factor that could lead to heterogeneity. Population-based controls could be more reliable than hospital-based controls because the genotype distributions in hospital-based controls may be deviated from normal. In this study, significant heterogeneity was found in three of the five polymorphisms. For these polymorphisms, the heterogeneity disappeared in subgroup analysis based on ethnicity, suggesting that ethnicity of the studied population are the major source of the heterogeneity.

The current study has some inevitable limitations that should be acknowledged. First, only published studies were included in this meta-analysis, which may have biased our results. Second, there was significant heterogeneity among included studies. Even though we used the random-effect model to calculate pool ORs, the precision of outcome would be affected. Third, our results were based on an unadjusted estimated, a more precise analysis would have been conducted if more detailed individual data were available.

In summary, we concluded that the MMP1-1607 1G/2G, MMP2-1306 C/T, MMP2-735 C/T, MMP9 -1562 C/T polymorphisms were risk factors for lung cancer among Asians, while MMP13 -77A/G polymorphism was not associated with lung cancer risk. However, future well designed large studies, particularly stratified by gene-gene and gene-environment interactions might be necessary to clarify the possible role of the MMP polymorphisms in the susceptibility to lung cancer.

## Additional Information

**How to cite this article**: Li, H. *et al.* Association of matrix metalloproteinase family gene polymorphisms with lung cancer risk: logistic regression and generalized odds of published data. *Sci. Rep.* **5**, 10056; doi: 10.1038/srep10056 (2015).

## Figures and Tables

**Table 1:** Characteristics of studies included in this meta-analysis.

**Author**	**Year**	**Country**	**Ethnicity**	**Source of control**	**Genotyping methods**	**Sample size (case/control)**		**Case**			**Control**		***P*_HWE_**
MMP1 -1607 1G/2G							1G/1G	1G/2G	2G/2G	1G/1G	1 G/2 G	2 G/2 G	
Biondi	2000	Italy	Caucasian	NA	NA	29/164	7	16	6	42	86	36	0.520
Su	2006	USA	Caucasian	PB	Taqman	2014/1323	541	1015	458	367	642	314	0.310
Zhang	2006	China	Asian	PB	PCR-RFLP	150/200	32	70	48	60	98	42	0.865
Gonzalez-Arriaga	2008	Spain	Caucasian	HB	PCR-RFLP	501/510	128	248	125	119	259	132	0.712
Hart	2011	Norway	Caucasian	PB	Taqman	436/434	115	207	114	132	198	104	0.081
Liu	2011	China	Asian	PB	PCR-RFLP	825/825	74	323	428	100	367	358	0.691
Fakhoury	2012	Lebanon	Caucasian	PB	PCR-RFLP	41/51	5	17	19	7	16	28	0.081
MMP2 -1306 C/T							CC	CT	TT	CC	CT	TT	
Yu	2002	China	Asian	PB	PCR-DHPLC	781/852	644	127	10	585	248	19	0.220
Zhou	2005	China	Asian	PB	PCR-RFLP	770/777	635	124	11	539	220	18	0.421
Rollin	2007	France	Caucasian	HB	PCR-RFLP	90/90	60	28	2	60	29	1	0.217
Song	2007	China	Asian	PB	PCR	163/148	129	32	2	100	44	4	0.747
Bayramoglu	2011	Turkey	Caucasian	HB	PCR-RFLP	200/100	123	73	4	65	32	3	0.692
MMP2 -735 C/T							CC	CT	TT	CC	CT	TT	
Zhou	2005	China	Asian	PB	PCR-RFLP	770/777	506	230	34	425	313	39	0.052
Rollin	2007	France	Caucasian	HB	PCR-RFLP	89/90	69	18	2	67	21	2	0.816
Jia	2009	China	Asian	PB	PCR-RFLP	370/436	260	96	14	292	123	21	0.092
MMP9 -1562C/T							CC	CT	TT	CC	CT	TT	
Zhang	2005	China	Asian	PB	PCR-RFLP	150/200	83	60	7	155	42	3	0.936
Rollin	2007	France	Caucasian	HB	PCR-RFLP	90/90	68	22	0	64	21	5	0.085
Bayramoglu	2009	Turkey	Caucasian	HB	PCR-RFLP	200/100	150	48	2	67	30	3	0.871
Gonzalez-Arriaga	2012	Spain	Caucasian	HB	PCR-RFLP	762/649	581	174	7	483	148	18	0.110
MMP13 -77A/G							AA	AG	GG	AA	AG	GG	
Gonzalez-Arriaga	2008	Spain	Caucasian	HB	PCR-RFLP	501/506	248	208	45	267	197	42	0.508
Peng	2010	China	Asian	PB	PCR-RFLP	420/419	105	207	108	91	227	101	0.085
Wang	2013	China	Asian	PB	PCR-RFLP	300/300	85	132	83	55	156	89	0.354

PB, Population–based; HB, Hospital–based; PCR-RFLP: Polymerase Chain Reaction-restriction Fragment Length Polymorphism; HWE: Hardy-Weinberg Equilibrium.

**Table 2:** Meta-analysis of MMP1,MMP2,MMP9 and MMP13 polymorphism and lung cancer risk.

**Studycharacteristics**		**Case/controls**	**Genetic model**	**OR(95%CI)**	***I*^*2*^ (%)**	***P* forheterogeneity**
MMP1 -1607 1G/2G						
Total (N = 7)		3996/3507	Additive Model	1.11(0.97-1.27)	64.2	0.010
			OR_G_	1.13(0.96-1.32)	63.4	0.012
Ethnicity	Caucasian(N = 5)	3021/2482	Additive Model	1.01(0.93-1.09)	0	0.656
			OR_G_	1.01(0.92-1.10)	0	0.652
	Asian(N = 2)	975/1025	Additive Model	1.34(1.18-1.53)	0	0.468
			OR_G_	1.41(1.21-1.65)	0	0.473
Source of control	PB(N = 5)	3466/2833	Additive Model	1.16(0.99-1.37)	70.9	0.008
			OR_G_	1.19(0.98-1.44)	70.2	0.009
	HB(N = 1)	501/510	Additive Model	0.94(0.79-1.12)	—	—
			OR_G_	0.93(0.75-1.14)	—	—
	NA(N = 1)	29/164	Additive Model	1.00(0.57-1.76)	—	—
			OR_G_	1.00(0.51-1.97)	—	—
Sample size	≥ 500(N = 4)	3776/3092	Additive Model	1.08(0.94-1.25)	74.4	0.008
			OR_G_	1.10(0.93-1.30)	73.8	0.010
	500(N = 3)	220/415	Additive Model	1.18(0.83-1.67)	39.2	0.193
			OR_G_	1.30(0.97-1.74)	42.8	0.174
MMP2 -1306 C/T						
Total (N = 5)		2004/1967	No association			
			OR_G_	0.64(0.46-0.87)	73.2	0.005
Ethnicity	Caucasian(N = 2)	290/190	OR_G_	1.09(0.74-1.59)	0	0.782
	Asian(N = 3)	1714/1777	OR_G_	0.49(0.42-0.57)	0	0.863
Source of control	PB(N = 3)	1714/1777	OR_G_	0.49(0.42-0.57)	0	0.863
	HB(N = 2)	290/190	OR_G_	1.09(0.74-1.59)	0	0.782
Sample size	≥ 500(N = 2)	1551/1629	OR_G_	0.48(0.41-0.57)	0	0.840
	500(N = 3)	453/338	OR_G_	0.84(0.63-1.15)	55.9	0.104
MMP2 -735 C/T						
Total (N = 3)		1229/1303	No association			
			OR_G_	0.72(0.62-0.84)	21.1	0.281
Ethnicity	Caucasian(N = 1)	89/90	OR_G_	0.85(0.44-1.67)	—	—
	Asian(N = 2)	1140/1213	OR_G_	0.71(0.61-0.84)	56.3	0.130
Source of control	PB(N = 2)	1140/1213	OR_G_	0.71(0.61-0.84)	56.3	0.130
	HB(N = 1)	89/90	OR_G_	0.85(0.44-1.67)	—	—
Sample size	≥ 500(N = 2)	1140/1213	OR_G_	0.71(0.61-0.84)	56.3	0.130
	500(N = 1)	89/90	OR_G_	0.85(0.44-1.67)	—	—
MMP9 -1562 C/T						
Total (N = 4)		1202/1039	Complete overdominant model	0.84(0.51-1.39)	79.1	0.002
			OR_G_	1.07(0.59-1.95)	87.0	< 0.001
Ethnicity	Caucasian(N = 3)	1052/839	Complete overdominant model	1.04(0.84-1.29)	0	0.568
			OR_G_	0.84(0.68-1.03)	0	0.600
	Asian(N = 1)	150/200	Complete overdominant model	0.40(0.25-0.64)	—	—
			OR_G_	2.73(1.74-4.27)	—	—
Source of control	PB(N = 1)	150/200	Complete overdominant model	0.40(0.25-0.64)	—	—
			OR_G_	2.73(1.74-4.27)	—	-
	HB(N = 3)	1052/839	Complete overdominant model	1.04(0.84-1.29)	0	0.568
			OR_G_	0.84(0.68-1.03)	0	0.600
Sample size	≥ 500(N = 1)	762/649	Complete overdominant model	1.00(0.78-1.28)	—	—
			OR_G_	0.89(0.70-1.13)	—	—
	500(N = 3)	440/390	Complete overdominant model	0.79(0.36-1.73)	83.3	0.003
			OR_G_	1.14(0.44-2.94)	89.7	<0.001
MMP13 -77 A/G						
Total (N = 3)		1221/1225	Recessive model	1.02(0.83-1.26)	0	0.711
			OR_G_	0.95(0.76-1.17)	57.8	0.094
Ethnicity	Caucasian(N = 1)	501/506	Recessive model	1.09(0.70-1.69)		
			OR_G_	1.12(0.90-1.41)		
	Asian(N = 2)	720/719	Recessive model	1.01(0.80-1.27)	0	0.446
			OR_G_	0.87(0.73-1.04)	39.6	0.198
Source of control	PB(N = 2)	720/719	Recessive model	1.01(0.80-1.27)	0	0.446
			OR_G_	0.87(0.73-1.04)	39.6	0.198
	HB(N = 1)	501/506	Recessive model	1.09(0.70-1.69)		
			OR_G_	1.12(0.90-1.41)		

OR_G_: The Generalized Odds Ratio.
